# Under simulated microgravity and gravity, anthocyanin is regulated by *DcaWRKY2* in *Dendrobium catenatum* leaves

**DOI:** 10.3389/fpls.2024.1505199

**Published:** 2025-01-20

**Authors:** Tianze Hou, Baoqiang Zheng, Fucheng Peng, Zehui Jiang, Wenbo Zhang, Yan Wang

**Affiliations:** ^1^ Key Laboratory of Tree Breeding and Cultivation of National Forestry and Grassland Administration, Research Institute of Forestry, Chinese Academy of Forestry, Beijing, China; ^2^ Key Laboratory of National Forestry and Grassland Administration/Beijing for Bamboo and Rattan Science and Technology, International Center for Bamboo and Rattan, Beijing, China

**Keywords:** anthocyanin, *Dendrobium catenatum*, *DcaWRKY2*, simulated microgravity, transcriptional regulation, leaves’ color

## Abstract

Long-term space missions will require high-quality plants that are edible, medicinal, and ornamental, to support the physical and mental health of astronauts under altered gravity conditions. Anthocyanins play a key role in enhancing the medicinal and edible value and ornamental properties of plants. However, under simulated microgravity, the transcription control of anthocyanin biosynthesis is not clear. Here, in order to investigate the influences of simulated microgravity on the anthocyanin accumulation further, clones of *Dendrobium catenatum* were exposed for 20 days to simulated microgravity conditions. The anthocyanin content in *Dendrobium catenatum* leaves increased in the simulated microgravity conditions compared with that in gravity-treated clones. Furthermore, based on the transcriptome sequencing, differentially expressed genes (DEGs), and weighted gene co-expression network analysis combined with RT-qPCR, we identified one WRKY gene, *DcaWRKY2*, from a *Dendrobium catenatum* under simulated microgravity conditions, which indicated that *DcaWRKY2* may be involved in anthocyanin biosynthesis under simulated microgravity conditions. A more in-depth analysis evaluating the function of *DcaWRKY2*, transcription factor gene *DcaWRKY2*, was silenced by virus-induced gene silencing under gravity conditions, which resulted in the increase of anthocyanin accumulation in leaves, and the expression levels of anthocyanin biosynthesis pathway (ABP) structural genes, including *DcaCHS*, *DcaCHI*, *DcaF3H*, *DcaDFR*, and *DcaANS* were increased significantly. This research provides new insights into how altered gravity can affect anthocyanin synthesis in plants and illuminated the regulatory effects of *DcaWRKY2* on the leaves’ pigmentation and anthocyanin biosynthesis in *Dendrobium catenatum* under gravity and simulated microgravity.

## 1 Introduction

The goal of long-term human exploration of space necessitates plant-based effective bioregenerative life support systems (BLSS) to produce edible biomass and oxygen, recycle water, absorb carbon dioxide and to support the physical and mental health of astronauts (Kordyum and Hasenstein, 2021). Plant growth and quality are the key factors of controlled BLSS, and a large number of space experiments have shown that the space microgravity environment seriously affects the growth and quality of plants, resulting in the impact of plant yield in space (Bula and Ignatius, 1996; Bugbee, 1999). It is of great significance to study and elucidate the mechanism of gravity on plant growth, development and quality for the establishment of a controlled BLSS. Plants on the earth are subject to gravity. Removing the effects of gravity on plants and then studying the changes in plants at the holistic, cellular, and molecular levels is an effective means to study the effects of gravity on plants. To remove the influence of gravity, the most ideal condition is the microgravity environment of space, which has been proved to undergo significant changes in the growth and development and metabolism level of plants at the individual level (Porterfield et al., 1997; Ueda et al., 2000). Among the various methods for simulating and generating microgravity (Obenland and Brown, 1994; Miyamoto et al., 1999), clinostats have been developed to partially simulate, in ground-based experiments, the weightless environment of space. Experiments have shown that the plants rotated in clinostats lose some of the characteristics of normal gravity and are very similar to some of the characteristics formed under space microgravity (Stanković et al., 2001; Miyamoto et al., 2005). Space mutagensis is a technology characterized with multiple orientations, high mutation frequency, safety, and high efficiency in industrial docking, which can be used to accelerate the breeding process and achieve germplasm enhancement for meeting the demand for special varieties. Simulated microgravity treatment is one of the space mutagensis, which shows that it is of great significance to carry out pioneering research work in the field of simulated microgravity.

*Dendrobium* species is famous for not only anti-aging effect, tumors, hyperglycemia, and hyperlipidemia (Ji et al., 2017; Wang et al., 2018a; Liang et al., 2019; Nie et al., 2020; Wang et al., 2022a; Wu et al., 2023) but also enhancing the body’s immunity, prolonging life, and resisting cancer (Takamiya et al., 2011). Moreover, *Dendrobium* species are widely used for ornamental industry. One of the most renowned species is *Dendrobium catenatum*, dwarfed, which has been widely cultivated worldwide due to its excellent medical, edible, and ornamental value. These advantages make it a good material for studying and elucidating plant growth and development and the synthesis of medicinal foods mechanism under microgravity.

As is widely acknowledged that anthocyanins are important flavonoid pigments and secondary metabolic compounds (Park et al., 2012) involved in the coloring of leaves and have been associated with beneficial effects concerning human health such as preventing obesity and type 2 diabetes (He and Giusti, 2010), anti-inflammatory and anticancer properties, and reducing the risk of cardiovascular diseases (Kruger et al., 2014). It is well established that anthocyanin biosynthesis is regulated by the MYB and bHLH transcription factors (TFs) in most plants under gravity conditions (Li et al., 2017). So far, under gravity conditions, several candidate genes that encode key enzymes involved in the anthocyanin biosynthesis pathway (ABP) such as *CHS*, *CHI*, *F3′ H*, *DFR*, and *ANS*, as well as the TF genes *DhMYB2* and *DhbHLH1* have been cloned, and the relationship between the gene expression and floral coloration in *Phalaenopsis*-type *Dendrobium* hybrids was investigated (Mudalige-Jayawickrama et al., 2005; Pitakdantham et al., 2011; Li et al., 2017; Hou et al., 2023). Secondary metabolites are of great value in breeding, such as stress resistance (Bennett and Wallsgrove, 1994; Bose et al., 2021; Wang et al., 2022b; Zhang et al., 2022), high yield (Gfeller et al., 2023; Pang et al., 2021; Khan et al., 2022), and ornamental traits (Noman et al., 2017; Xiao et al., 2022; Mostafa et al., 2022; Du et al., 2022). Because secondary metabolic compounds are so important in determining the nutritional and quality of plants, their responses to altered gravity are now being studied (Tuominen et al., 2009). However, under simulated microgravity, changes in anthocyanin content in plants have not been reported at the cellular level and the transcription control of anthocyanin biosynthesis in *Dendrobium catenatum* leaves at the molecular level under simulated microgravity is not clear. Here, in order to investigate the influences of simulated microgravity on the anthocyanin accumulation further, clones of *Dendrobium catenatum* were exposed for 20 days to simulated microgravity conditions. The anthocyanin content in *Dendrobium catenatum* leaves increased in the simulated microgravity conditions compared with gravity-treated clones. Furthermore, based on the transcriptome sequencing, DEG and weighted gene co-expression network analysis (WGCNA) combined with Real-time fluorescence quantitative PCR (RT-qPCR), we identified one WRKY gene, *DcaWRKY2*, from a *Dendrobium catenatum* under simulated microgravity conditions, which indicated that *DcaWRKY2* may be involved in anthocyanin biosynthesis under simulated microgravity conditions. A more in-depth analysis evaluating the function of *DcaWRKY2*, TF gene *DcaWRKY2*, was silenced by virus-induced gene silencing (VIGS) under gravity conditions, which resulted in the increase of anthocyanin accumulation in leaves, and the expression levels of ABP structural genes, including *DcaCHS*, *DcaCHI*, *DcaF3H*, *DcaDFR*, and *DcaANS*, were increased significantly. This research provides new insights into how altered gravity can affect anthocyanin synthesis in plants and illuminated the regulatory effects of *DcaWRKY2* on the leaves’ pigmentation and anthocyanin biosynthesis in *Dendrobium catenatum* under gravity and simulated microgravity.

## 2 Materials and methods

### 2.1 Plant materials

Strong biennial clones of *Dendrobium catenatum* were grown in a controlled-environment room (12/12-h light/dark, light intensity ~100 μmol m^−2^ s^−1^; 28°C/22°C day/night; and relative humidity 60/70% day/night) and adapted to the controlled conditions for 10 days before being used for the follow-up experiment.

### 2.2 Clinostat treatments

A 3-D clinostat (SM-31 two-axis driving clinostat) was used to simulate weightlessness. Characteristic physical details of this instrument were given by Wei et al. (2010) with a little modification. Briefly, this device was designed as a group of orthogonal axes allowing simultaneous rotation around each of two horizontal and vertical axes. The rotation rate of the clinostat (0.5–2.5 rpm) could be adjusted and controlled by a control box. The clinostat rotation treatment in this study consisted of a clinostat oriented horizontally and rotating at 1.5 rpm (e.g., the level of acceleration applied to the materials was less than 2.8 × 10^−3^ g at a radius of 2.5 cm) (Khai et al., 2021). In addition to the stationary 1 g control, a vertically oriented clinostat rotating at 1.5 rpm was used as a control to evaluate potential mechano-stimulation artefacts from the clinostat motor and the side-effects of rotation itself. Six digital cameras for visible image acquisition were installed in the 3-D clinostat, allowing the recording of plants every 120 min.

### 2.3 Illumina cDNA library construction, sequencing, and data analysis

Samples collected at 0 day, 10 days of control check under gravity, 10 days of treatment under simulated microgravity, 15 days of control check under gravity, and 15 days of treatment under simulated microgravity were chosen for transcriptome sequencing according to the morphological characteristics and changes in total anthocyanin content during 20 days of simulated microgravity treatment of *Dendrobium catenatum*. Total RNA of the leaves was extracted using TRIzol Reagent according the manufacturer’s instructions (Invitrogen, USA), and the quality was assessed on the Nanodrop and Agilent 5300 system. Subsequently, the library from three biological sample replicates was prepared following Illumina^®^ Stranded mRNA Prep, Ligation (San Diego, CA) using 1 μg of total RNA, which was then sequenced with the NovaSeq X Plus Platform (2× 150-bp read length) supplied by Shanghai Majorbio Bio-pharm Technology Co., Ltd. (China). The raw paired-end reads were trimmed and quality-controlled by fastp (Chen et al., 2018) with default parameters. Then, clean reads were separately aligned to *Dendrobium catenatum* reference genome with orientation mode using HISAT2 (Kim et al., 2015) software. The mapped reads of each sample were assembled by StringTie (Pertea et al., 2015) in a reference-based approach. The expression level of each gene was calculated by using RNA-Seq by Expectation-Maximization (RSEM) and converted into fragments per kilobase per million fragments (FPKM) according to the read counts (Li and Dewey, 2011).

### 2.4 Differential gene analysis

Differential expression analysis was performed using DESeq2. The threshold for significantly differentially expressed genes (DEGs) was set at a |log2(fold change)| ≥1 and p adjust <0.05. Clustering analysis of DEGs was performed by R statistical package. The Euclidean distance was used to perform hierarchical clustering analysis on 7,562 and 3,002 differential genes, which differentially expressed in leaves between 10d T vs. 10d CK (10 days of treatment under simulated microgravity vs. 10 days of control check under gravity) and 15d T vs. 15d CK (15 days of treatment under simulated microgravity vs. 15 days of control check under gravity), and the clusters positively correlated with the change of anthocyanin content were selected as key candidate clusters. Based on this, further TF analysis was carried out.

### 2.5 Gene co-expression network analysis

Hub genes highly involved in anthocyanin pathway were identified using WGCNA. A total of 8,619 genes were obtained in 15 *Dendrobium catenatum* samples after screening out the genes with mean FPKM value and variable coefficient less than 1 and 0.1, respectively, and further utilized for co-expression network construction by WGCNA software (version 1.63). Modules were identified by setting the following parameters: a soft power β of 12, a minimum model size of 30, and a merge cut height of 0.22. Drawing from this foundation, a more in-depth analysis of TFs was subsequently initiated.

### 2.6 Virus-induced gene silencing of *DcaWRKY2* in *Dendrobium catenatum*

A 123-bp fragment of *DcaWRKY2* (1–123 bp) was amplified and cloned into the pTRV2-GFP vector (modified from pTRV2 vector) to construct the pTRV2-*DcaWRKY2*-GFP recombinant vector. The primers are listed in **Supplementary Table S1**. Then, the pTRV1, pTRV2-GFP, and pTRV2-*DcaWRKY2*-GFP plasmids were transformed into the A. strain GV3101, which was cultured in the LB medium [kanamycin, 100 mg/L; rifampicin, 25 mg/L; 200 μM Acetosyringone (AS); and 10 mM MESmonohydrate (MES)] at 28°C for 13–15 h until an Optical Density at 600 Nanometers (OD600) = 1.7 was reached. After centrifugation, the thallus was resuspended until reaching OD600 = 1.0 in an infiltration buffer containing 10 mM MES, 100 μM AS, and 10 mM MgCl_2_. Finally, pTRV1 was mixed with pTRV2-GFP and pTRV2-*DcaWRKY2*-GFP in equal volumes, respectively, and the mixed bacterial solution was allowed to stand for 3.5 h at room temperature in the dark (Han et al., 2022; Liu et al., 2002; Chen et al., 2004; Burch-Smith et al., 2006; Jiang et al., 2011; Quadrana et al., 2011; Bachan and Dinesh-Kumar, 2012). Clones of *Dendrobium catenatum* were cultivated in white and transparent pots (5.0 cm in diameter) with sphagnum moss as the matrix. The plants were grown in a greenhouse with temperatures from 22°C to 28°C and relative humidity from 40% to 60%. The experimental treatment time was in mid-May, and the experiments were initiated with strong annual clones grown in matrix maintained at approximately 30% volumetric water content, which ensured that these plants did not undergo drought or waterlogging. The injection experiment was carried out in the afternoon (15:00–17:00) with good weather conditions. Suspensions (300 μL) of each was injected into the leaf according to Hou et al. (2023) by use of a 1-mL syringe in all VIGS treatments. The infiltrated leaves were given shaded treatment for 24 h and then allowed to grow normally in a natural state. After 7–10 days, photographs were taken, and leaves were collected for subsequent experiments. The leaves of *Dendrobium catenatum* exhibiting silencing phenotype treated by *DcaWRKY2*-VIGS as well as the untreated leaves were checked for the existence or absence of pTRV1 and pTRV2-*DcaWRKY2* by detecting the TRV vectors. RT-PCR was performed using the primer pairs TRV1-F, TRV1-R, TRV2-F, and TRV2-R.

### 2.7 Determination of anthocyanin content

The fresh *Dendrobium catenatum* leaves were collected from the plants after days 0, 5, 10, 15, and 20 under gravity and simulated microgravity and *DcaWRKY2*-silenced plants that showed VIGS phenotypes for each treatment at 7 days past infiltration (dpi) under gravity. Total anthocyanin was extracted from the fresh *Dendrobium catenatum* leaves by 0.1% HCl in methanol. Chloroform was used to remove chlorophylls in the extraction of leaves. In addition, the filtered extraction was used for determination of absorbance at 525 nm. Anthocyanin contents were expressed in mg/g fresh weight of standard cyanidin 3-rutinoside (Li et al., 2017).

### 2.8 Real-time quantitative RT-qPCR

The third and fourth mature leaves from the apex of each individual exhibiting VIGS phenotypes were collected from individual *DcaWRKY2*-silenced plants at 7 dpi. The same sized leaves from wild plants with no agroinfiltration treatment were taken as the control. Total RNA was extracted from the whole leaves by use of the RNA prep Pure Plant Plus Kit (Polysaccharides Polyphenolics-rich) (TIANGEN BIOTECH Co. Ltd., Beijing, China) and treated with Ribonuclease (RNase)-free Deoxyribonuclease I (DNase) (TIANGEN BIOTECH) to remove residual DNA. cDNAs were synthesized using reverse transcriptase using the HiScript III RT SuperMix for qPCR (+gDNA wiper) (Vazyme BIOTECH Co. Ltd., Nanjing, China). The primers used for RT-qPCR amplification are listed in **Supplementary Table S1**. Reactions involved incubation at 95°C for 7 min and thermal-cycling for 40 cycles (95°C for 5 s and 60°C for 30 s). The RT-qPCR results were calculated using the 2^−ΔΔCq^ method.

## 3 Results

### 3.1 Clinostat rotation conditions resulted in color increase in the *Dendrobium catenatum* leaves

**Figure 1:**
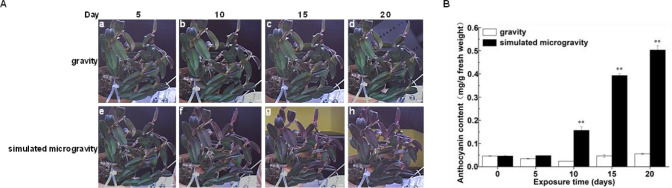
Time-course series of digital images showing the growth and anthocyanin content of *Dendrobium catenatum* under gravity and simulated microgravity. **(A)** Example images of plants were recorded. a, day 5 under gravity; b, day 10 under gravity; c, day 15 under gravity; d, day 20 under gravity; e, clinostat day 5; f, clinostat day 10; g, clinostat day 15; h, clinostat day 20. Bar, 1 cm. **(B)** The content of total anthocyanin in the leaves under gravity and simulated microgravity plants. And the anthocyanin was extracted from the freshly leaves after days 0, 5, 10, 15, and 20 under gravity and simulated microgravity. Data are mean ± SD (n = 3). The ** above the bars are statistically different by Duncan’s Multiple Range Test (p < 0.01).

In this study, clones of *Dendrobium catenatum* exposed for 20 days to simulated microgravity conditions. Leaves exhibited increasing of leaves’ colors under clinostat rotation conditions. These leaves’ color phenotypes become increasingly clearer in the successively simulated microgravity conditions. Relative to the leaves under gravity conditions, the leaves under 10 days of clinostat rotation conditions showed increase colors (**Figures 1A–f**). Under 15 days of clinostat rotation conditions, the leaves showed the more intense (**Figures 1A–g**). The leaves, under 20 days of clinostat rotation conditions, had extremely increase colors (**Figures 1A–h**). In particular, the leaves, under 10 and 15 days of clinostat rotation conditions displayed a substantial increase in total anthocyanin content (**Figure 1B**). Thus, the leaves under 0, 10, and 15 days of clinostat rotation conditions were selected to transcriptome analysis in the following study of the transcription control of anthocyanin biosynthesis under simulated microgravity.

### 3.2 Transcriptome sequencing and analysis

**Figure 2:**
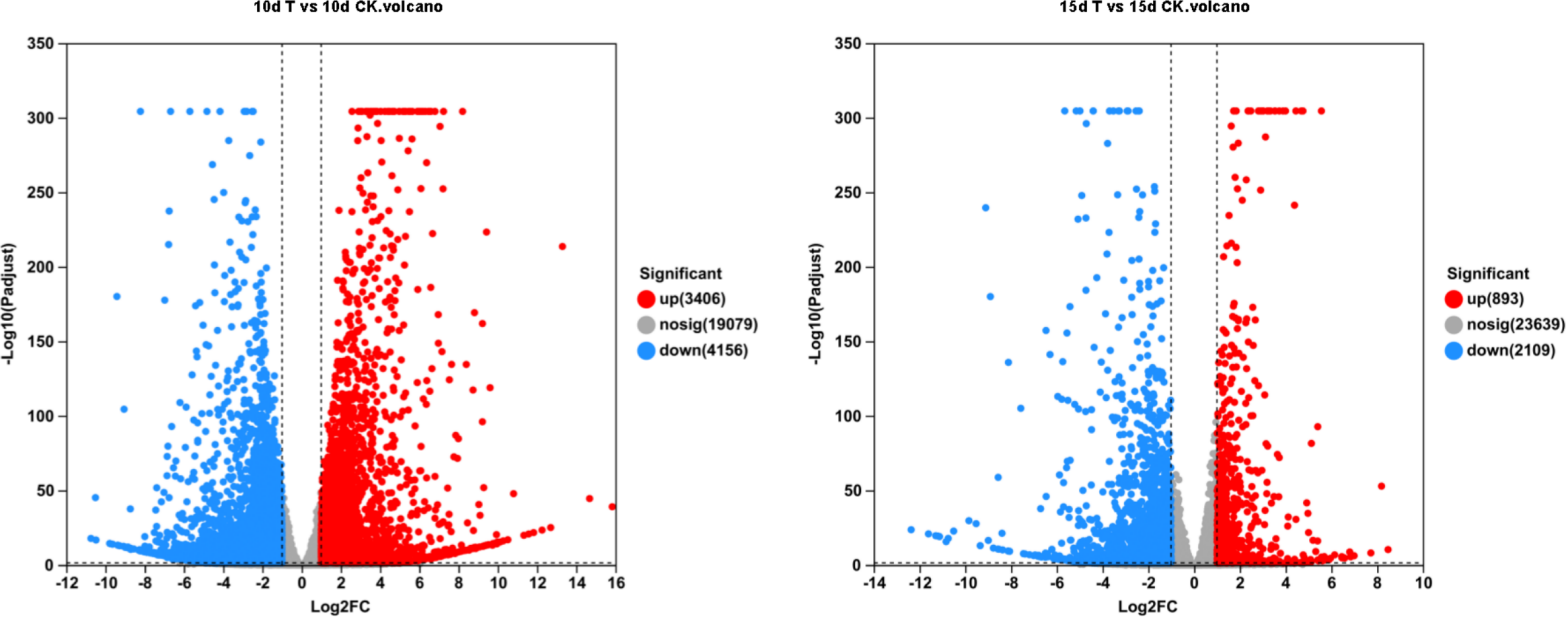
Volcanic plot of *Dendrobium catenatum* differentially expressed genes identified by transcriptome analysis of 10d T vs. 10d CK and 15d T vs. 15d CK. Raw counts were directly analyzed by DESeq2 software based on negative binomial distribution, and genes with expression differences between groups were obtained on the basis of certain standardized processing and screening conditions, and the default parameters were P adjust <0.05 and |log2FC| ≥1.

**Figure 3:**
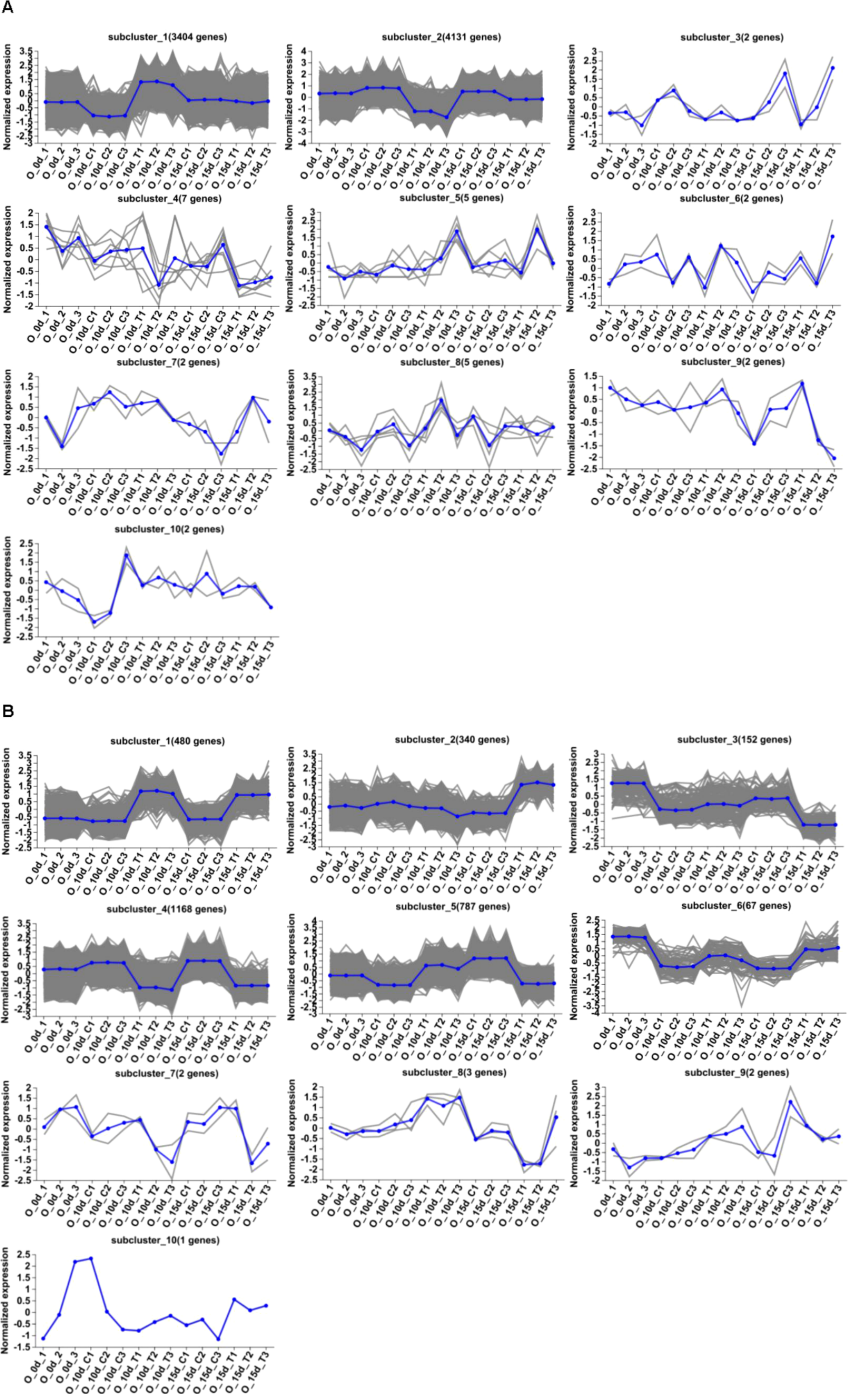
Hierarchical clustering analysis. **(A)** Hierarchical clustering analysis of 7,562 DEGs identified from 10d T vs. 10d CK. A total of 10 clusters were identified on the basis of the expression profiles of these genes. **(B)** Hierarchical clustering analysis of 3,002 DEGs identified from 15d T vs. 15d CK. A total of 10 clusters were identified on the basis of the expression profiles of these genes.

**Figure 4:**
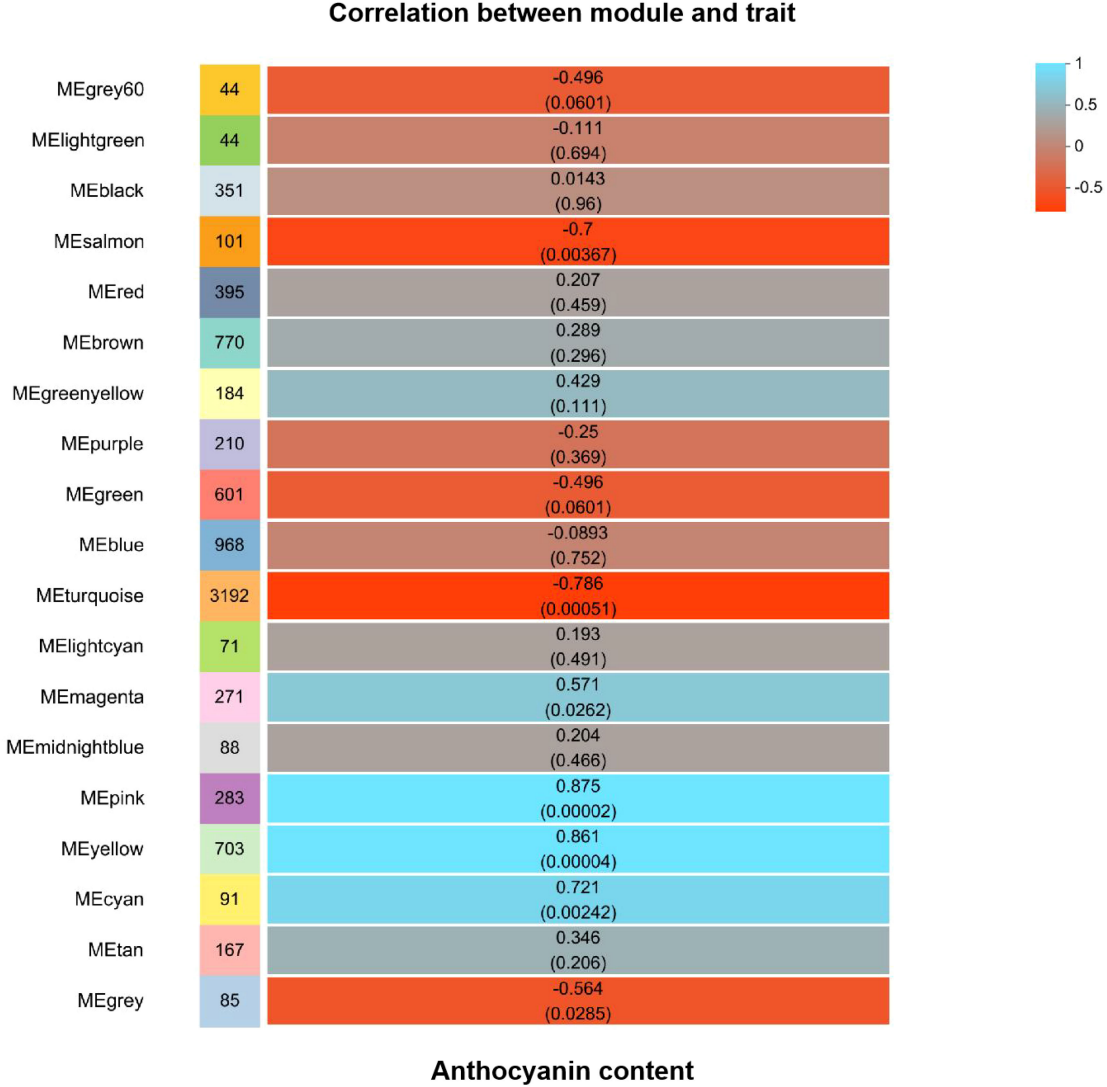
Module-anthocyanin content correlations with corresponding P-values. The color scale of squares indicates module–trait correlation from −1 (red) to 1 (blue).

**Figure 5:**
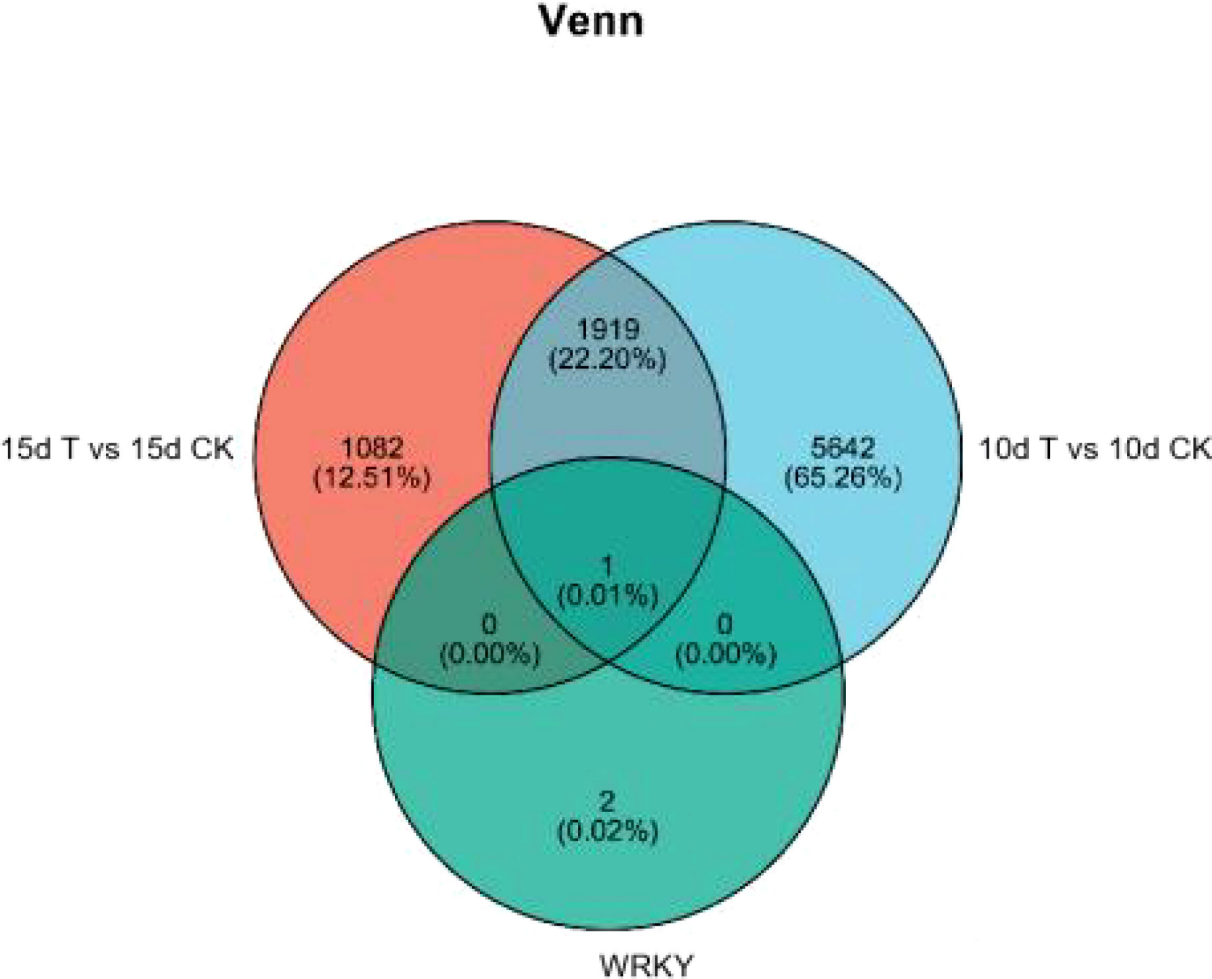
Analysis of WRKY transcription factors in 15d T vs. 15d CK and 10d T vs. 10d CK and WGCNA module turquoise.

Differential genes were analyzed for 10d T vs. 10d CK and 15d T vs. 15d CK (**Figure 2**). Through hierarchical clustering analysis, compared with the control, it was found that some subclusters were quite different after treatment. Among them, 7,562 differential genes from 10d T vs. 10d CK were divided into 10 clusters, the differentially changing genes and anthocyanins were negatively correlated in cluster 2, 3,002 differential genes from 15d T vs. 15d CK were divided into 10 clusters, and there was a negative correlation between differentially changing genes and anthocyanins in cluster 4 (**Figure 3**). The analysis showed that a common TF, *DcaWRKY2* (*Dca028004*), was different in the two comparison groups, and both were downregulated after treatment compared with the control. Then, the WGCNA was done. Among these modules, the MEturquoise module of 3,192 genes was highly negatively correlated with anthocyanin contents (r = −0.786, P = 5.1 × 10^−4^) (**Figure 4**). WRKY TF of MEturquoise module was further analyzed. The subsequent Venn analysis found that the TF that was the same difference under the 15d T vs. 15d CK and 10d T vs. 10d CK was *DcaWRKY2* (*Dca028004*), which indicated that it may be used as a WRKY TF to regulate anthocyanins (**Figure 5**).

### 3.3 Validation of gene expression by RT-qPCR

**Figure 6:**
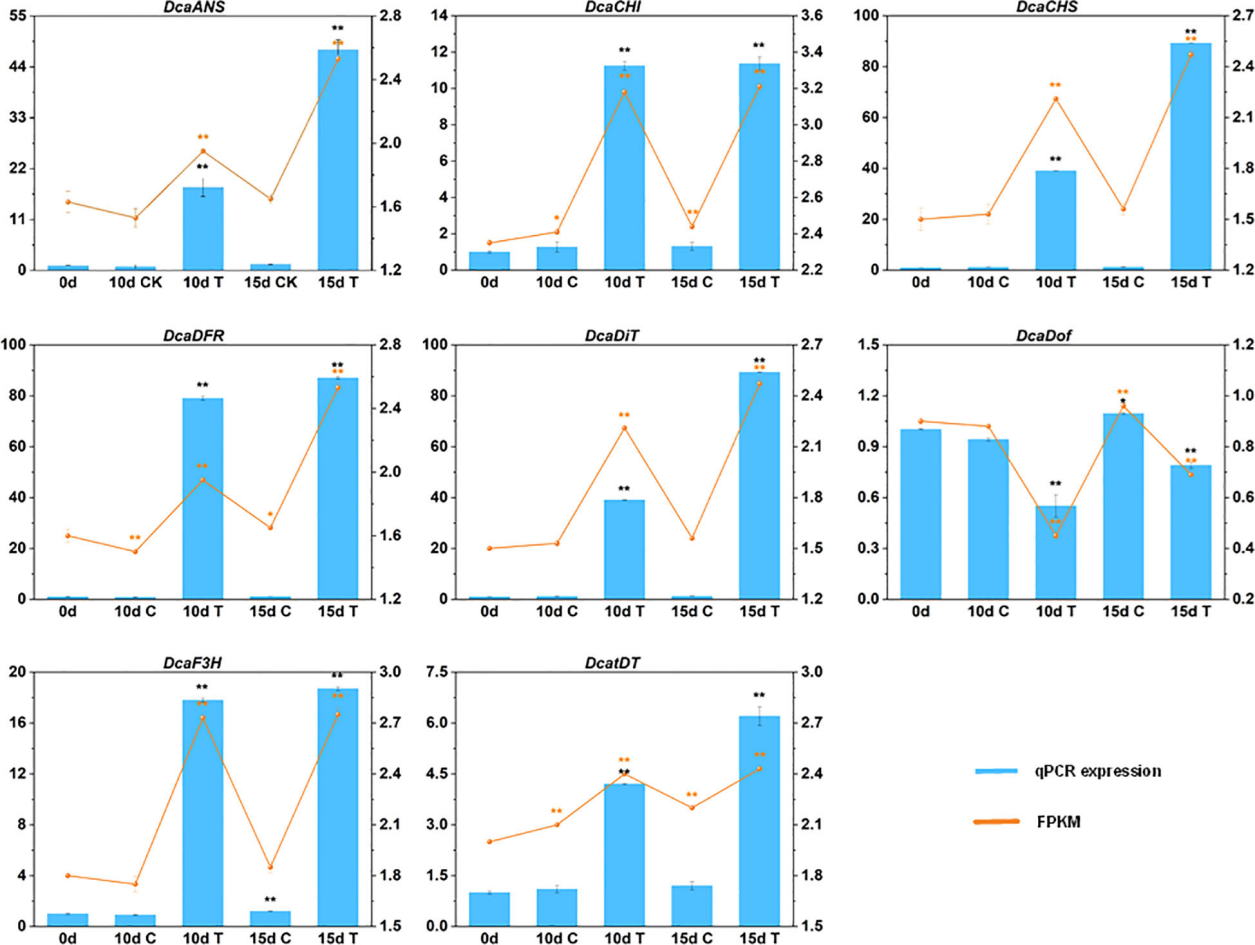
The expression patterns of eight selected genes identified by RNA-seq were verified by RT-qPCR. Data are mean ± SD (n = 3). The ** and * above the bars are statistically different by Duncan’s multiple range test (*p* < 0.01 and *p* < 0.05, respectively).

To confirm the credibility of our RNA sequencing (RNA-seq) data, the expression of eight DEGs were selected for RT-qPCR assays. The variation trends of these eight genes at different treatment points showed a high degree of consistency with the change tendency of the transcript FPKM values (**Figure 6**). The high congruence between the RNA-seq and RT-qPCR results indicated the reliability of the gene expression values in our experiment.

### 3.4 Verification of VIGS of *DcaWRKY2* in *Dendrobium catenatum* leaves

**Figure 7:**
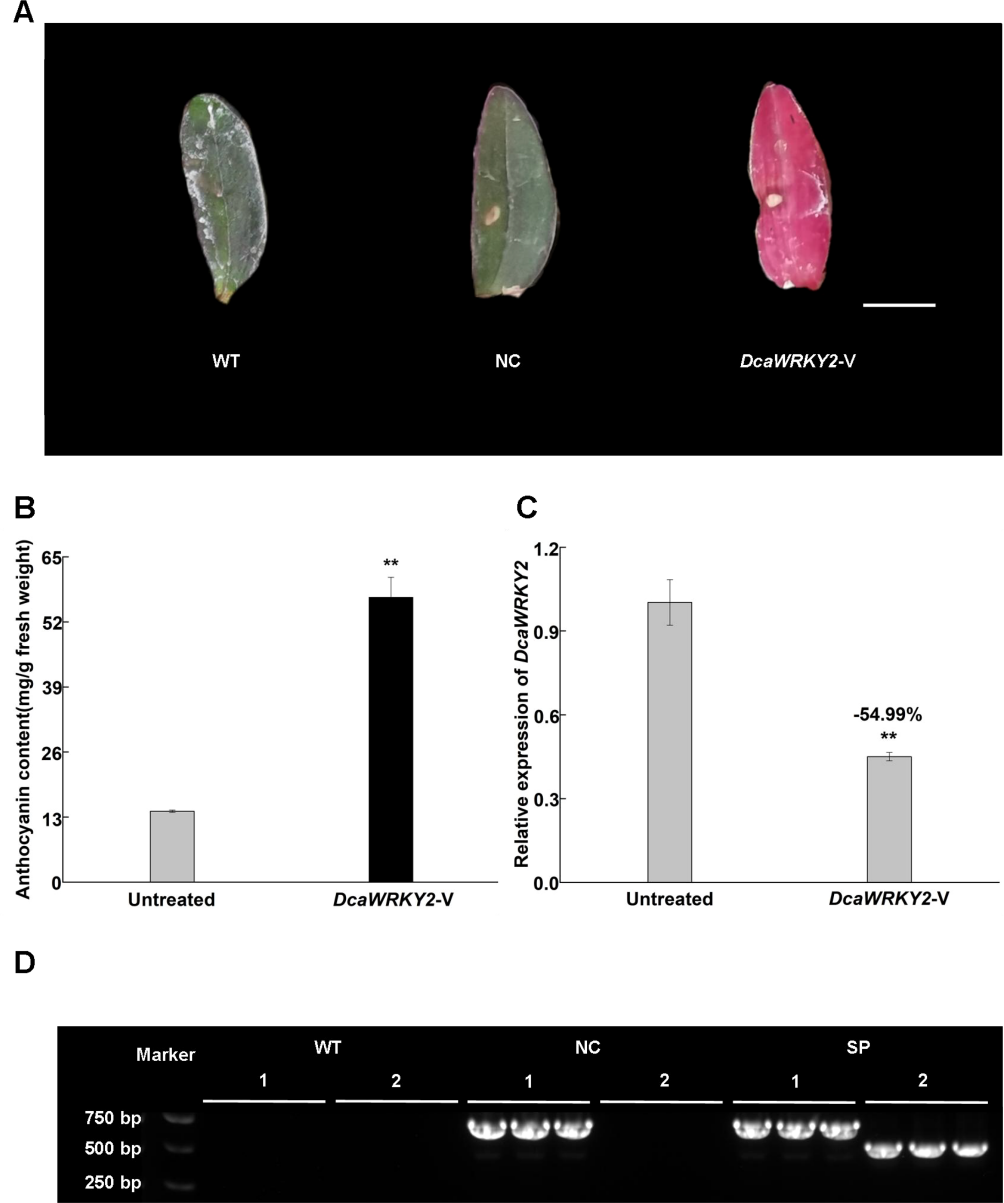
Validation of VIGS in *Dendrobium catenatum*. **(A)** Representative leaves from untreated plants and *DcaWRKY2*-VIGS–treated plants, WT: blank control without treatment; NC, negative control, infected with the empty vector, *DcaWRKY2*-V: *DcaWRKY2*-VIGS–treated plants. Bar, 1 cm. **(B)** The content of total anthocyanin in the leaves in untreated and *DcaWRKY2*-V treated plants. **(C)** Relative expression of *DcaWRKY2* of leaves in untreated and *DcaWRKY2*-silenced plants. The total RNA was extracted from the mature leaves at 7 dpi. In addition, the anthocyanin was extracted from the freshly leaf tissues at 7 dpi. Data are mean ± SD (n = 3). The ** above the bars are statistically different by Duncan’s multiple range test (*p* < 0.01). **(D)** Detection of gene expression in inoculated *Dendrobium catenatum.* 1, pTRV1; 2, pTRV2-*DcaWRKY2*. Data are mean ± SD (n = 3). No presence of pTRV1 and pTRV2-*DcaWRKY2* in WT, and the presence of only pTRV1 in NC, whereas both were present in pTRV1 and pTRV2-*DcaWRKY2* in silencing phenotype plants (SP).

To further explore the biological function of *DcaWRKY2*, *Dendrobium catenatum* with green leaves were infiltrated with *DcaWRKY2*-V treatment. The results showed that the leaves of *DcaWRKY2*-V–treated plants had begun to show relatively an extensive increase in color after approximately 7 dpi compared with the untreated control (**Figure 7A**). The increased leaves’ color produced by *DcaWRKY2*-V treatments was caused by the increase of total anthocyanin content. As shown in **Figure 7B**, the anthocyanin accumulation increased significantly in leaves after VIGS treatments.

### 3.5 The silencing of *DcaWRKY2* in *Dendrobium catenatum* leaves

To further verify whether the *DcaWRKY2* was silenced, the mRNA level of *DcaWRKY2* after VIGS treatments in leaves showing color increase was measured using RT-qPCR. The transcripts of *DcaWRKY2* were decreased in *DcaWRKY2*-V–treated leaves compared with the untreated leaves (**Figure 7C**), with an expression level reduced by ca. 55%. RT-PCR showed that the presence of pTRV1 was detected in both negative control (NC; infecting pTRV2 empty vector and pTRV1) and *DcaWRKY2*-silenced leaves, whereas pTRV2-*DcaWRKY2* was only detected in *DcaWRKY2*-silenced leaves and was absent in noninfiltrated control (WT; no treatment) (**Figure 7D**). These results confirmed that TRV vectors could infiltrate *Dendrobium catenatum* and that pTRV2-*DcaWRKY2* successfully induced transient gene knockdown to exhibit increase of leaves’ color phenotypes.

### 3.6 The expression of major genes in the ABP was increased by *DcaWRKY2* silencing

**Figure 8:**
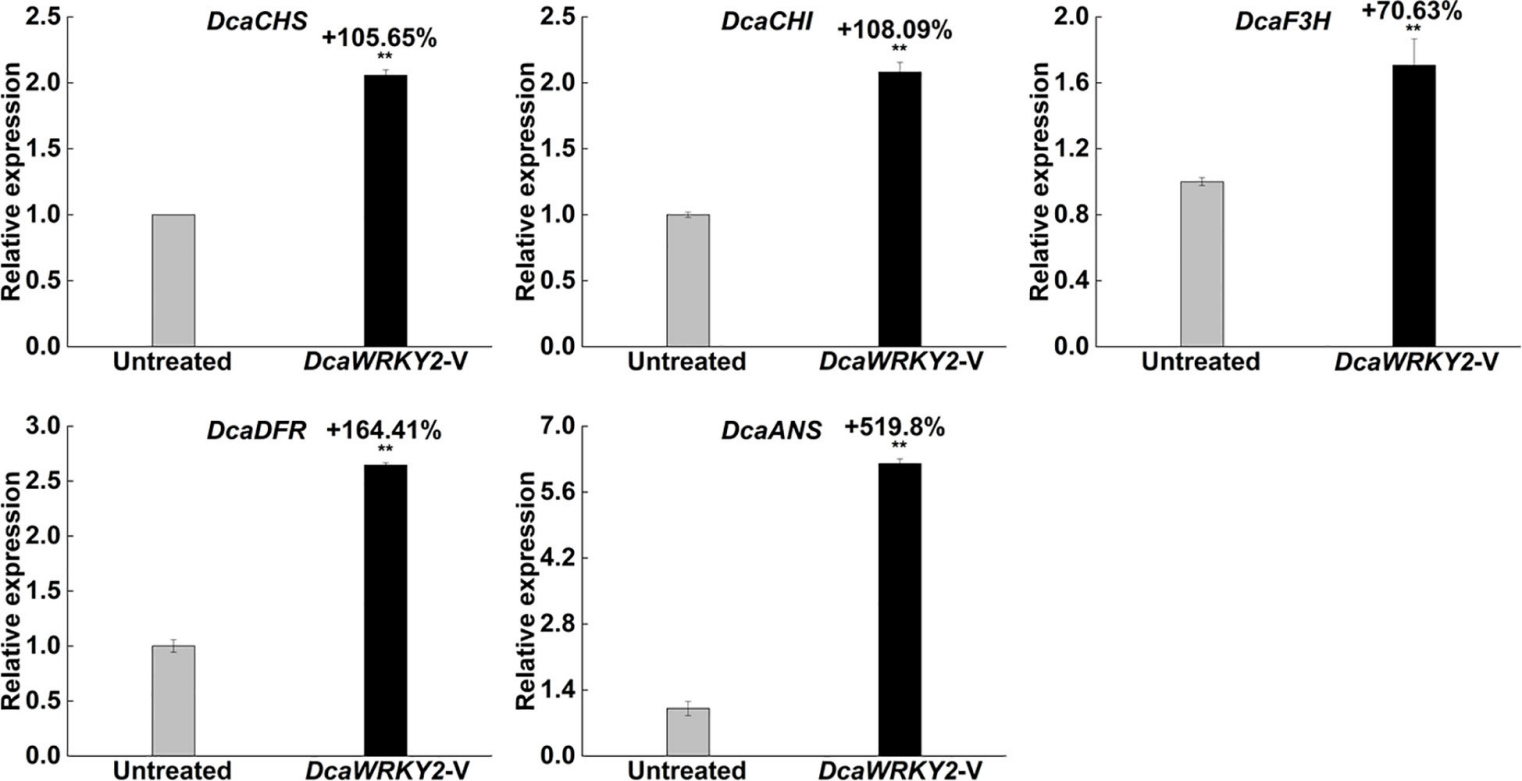
Relative expression of the structural genes on anthocyanin biosynthesis pathway and the transcription factor gene *DcaWRKY2*-V–treated leaves at 7 dpi. Data are mean ± SD (n = 3). The ** above the bars are statistically different by Duncan’s multiple range test (*p* < 0.01).

To detect the effect of *DcaWRKY2* silencing on the key genes of the ABP, their expression levels in the leaves were measured using RT-qPCR. The results suggested that the transcription levels of a series of enzyme genes involved in anthocyanin biosynthesis were increased to varying degrees in *DcaWRKY2*-silenced leaves (**Figure 8**). The relative expression of the early biosynthetic genes, *DcaCHI* and *DcaF3H*, as well as the late biosynthetic genes, *DcaDFR* and *DcaANS*, was 70.63% to 519.8% higher in leaves after the *DcaWRKY2*-V treatment.

## 4 Discussion

Anthocyanin content changes in *Dendrobium catenatum* leaves under simulated microgravity. The characterization of metabolic modification is generally considered to be one of the most direct approaches for studying the physiological growth, development, and quality changes in plant subjected to clinostat rotation conditions (Obenland and Brown, 1994; Soga et al., 2001). The response of secondary metabolic compounds to altered gravity was mostly concentrated in studies isoflavonoid and lignin (Cowles et al., 1984; Allen et al., 2009; Grudzińska et al., 2024). There is still no research on the changes of anthocyanin content in plants under simulated microgravity. The present study first reported that the clones of *Dendrobium catenatum* exposed for 20 days showed an increase of anthocyanin content and changes in leaves’ color relative to gravity-treated clones. Anthocyanins are very beneficial to human health and they can inhibit and prevent the growth of a variety of cancers under gravity, such as leukemia (Tsai et al., 2014) and lung cancer (Aqil et al., 2012). In addition, undergravity, anthocyanins have anti-inflammatory (Akiyama et al., 2012), anti-cardiovascular disease (Wallace, 2011), anti-obesity and diabetes (Muraki et al., 2013), and improving vision (Matsumoto et al., 2001) effects. The results of the current study may be useful in the future for disease treatment and plant variety improvement. The anthocyanins added in *Dendrobium catenatum* leaves under simulated microgravity can be used in the future for the treatment of diseases such as cancer and diabetes, as well as for the variety improvement of *Dendrobium catenatum. Dendrobium* species and anthocyanins have similar effects, such as anti-cancer (Takamiya et al., 2011; Ji et al., 2017; Wang et al., 2018a; Liang et al., 2019; Nie et al., 2020; Wang et al., 2022a; Wu et al., 2023). Therefore, it is speculated that the medical and edible quality of *Dendrobium catenatum* after microgravity treatment is better. On the other hand, the changes in leaf color improves the ornamentation and will be more beneficial to the physical and mental health of astronauts on the space station in the future. In lily, improvement of flower color by means of leaf treatments (Burchi et al., 2010). *UGT75C1* was significantly enriched in the ABP and had an effect on the color of *Oncidium* flowers and leaves (Wang et al., 2023). The flowers of *Dendrobium catenatum* are now yellow-green, and, if the leaf color change of *Dendrobium catenatum* after simulated microgravity treatment will lead to a change in flower color, then it will increase the ornamentality more.

TF *DcaWRKY2* involved in anthocyanin synthesis under gravity and microgravity. The effect of WRKY on plant anthocyanin metabolism under altered gravity has not been previously reported. Previous studies on changes in secondary metabolite content under altered gravity conditions have only been at the cellular level, and the molecular mechanisms underlying the formation and regulation of these metabolites have not been studied at the molecular level (Cowles et al., 1984; Allen et al., 2009; Grudzińska et al., 2024). In this experiment, the *DcaWRKY2* gene screened under microgravity conditions was verified and found to regulate the synthesis of anthocyanins under gravity, which showed that the function of *DcaWRKY2* was responsible for the synthesis of anthocyanins under gravity and microgravity. For the first time, we explored the phenotype of increased anthocyanin content in *Dendrobium catenatum* leaves under simulated microgravity and innovatively explored the molecular mechanism of anthocyanin formation and regulation in *Dendrobium catenatum* leaves under gravity and simulated microgravity at the molecular level. Previous studies have focused on WRKY involvement in the regulation of anthocyanin metabolism under gravity conditions. Experimental evidence has shown that *AtMYB75*, *AtMYB111*, and *AtMYBD* positively regulate the gene expression of anthocyanins in *Arabidopsis wrky41* mutants, resulting in a significant increase in anthocyanin content (Duan et al., 2018). Overexpression of *GbWRKY1* in *Arabidopsis thaliana* significantly reduced the accumulation of anthocyanin glycosides under low phosphorus stress (Bakshi and Oelmüller, 2014). The content of anthocyanin glycosides in the RNAi mutant of *Arabidopsis thaliana AtWRKY75* was five-fold higher than that of the wild type (Devaiah et al., 2007). However, under gravity conditions, no studies have shown that *WRKY2* gene has the function of regulating anthocyanin synthesis. Our experimental results show for the first time that *DcaWRKY2* can regulate the synthesis of anthocyanins under gravity.

*DcaWRKY2* silencing caused the increased expression of major genes in the ABP under gravity. Under simulated microgravity conditions, no genes have been found for *WRKY2* to regulate the ABP pathway. Under gravity conditions, it has been shown that WRKY regulates genes on the ABP pathway. Overexpression of *MdWRKY11* in apple calli can promote the expression of flavonoid synthesis structural genes such as *F3H*, *FLS*, *DFR*, *ANS*, and *UFGT* and promote the accumulation of flavonoids and anthocyanin glycosides in callus (Wang et al., 2018b). The Apple *MdWRKY41* or *MdWRKY41*-*MdMYB16* complex inhibits the accumulation of anthocyanins and proanthocyanidins by inhibiting the expression of *MdMYB12*, *MdLAR*, *MdUFGT*, and *MdANR* (Mao et al., 2021). In petunias, mutants of the WRKY gene *ph3* showed an increase in vacuolar pH and hindered anthocyanin accumulation in petals, and quantitative analysis showed a more than three-fold decrease in *DFR*, the structural gene for anthocyanin synthesis (Verweij et al., 2016). In pears, *PbWRKY75* induces anthocyanin synthesis in pear peel by activating the expression of *PbMYB10b* and the anthocyanin late biosynthesis genes *PbDFR* and *PbUFGT* (Cong et al., 2021). However, under gravity conditions, no studies have shown that WRKY2 regulates genes on the ABP pathway. In this study, we identified *DcaWRKY2* from *Dendrobium catenatum* leaves under simulated microgravity and *DcaWRKY2* was silenced by VIGS under gravity conditions, which resulted in the increase of anthocyanin accumulation in leaves, and the expression levels of ABP structural genes were increased significantly. We speculate that *DcaWRKY2* is the crucial WRKY TF regulating the full anthocyanin pigmentation in the leaves in *Dendrobium catenatum* under gravity and simulated microgravity.

## 5 Conclusion

This study isolated one WRKY gene, *DcaWRKY2*, from the *Dendrobium catenatum* leaves’ RNA-seq database under simulated microgravity. We successfully silenced *DcaWRKY2* by VIGS, and gene expression analysis in silenced leaves demonstrated that *DcaWRKY2* could positively regulate anthocyanin biosynthesis under gravity. These results suggested that *DcaWRKY2* play a role in regulating anthocyanin production in *Dendrobium catenatum* leaves under gravity and simulated microgravity. This study provides valuable information on anthocyanin biosynthesis regulation in *Dendrobium catenatum* under gravity and simulated microgravity.

## Data Availability

The data presented in the study are deposited in the National Center for Biotechnology Information Sequence Read Archive (SRA), accession number PRJNA1207207.
