# Supplementary material for: Under simulated microgravity and gravity, anthocyanin is regulated by DcaWRKY2 in Dendrobium catenatum leaves
Source: Front Plant Sci. 2025 Jan 20;15:1505199. doi: 10.3389/fpls.2024.1505199 (PMC11788367; doi:10.3389/fpls.2024.1505199)
Supplement: Supplementary file 1 [file DataSheet1.docx]

# Supplementary Data

Table S1. Primers used in the present study.

| Primer name | Primer sequences (5′-3′) |
| --- | --- |
| DcaWRKY2-pTRV2-F | GTGAGTAAGGTTACCGAATTCATGGCTGGGATGGATGACG |
| DcaWRKY2-pTRV2-R | CGTGAGCTCGGTACCGGATCCTGGCTCTGATAGAGAACCATCGT |
| TRV1-F | TTACAGGTTATTTGGGCTAG |
| TRV1-R | CCGGGTTCAATTCCTTATC |
| TRV2-F | CTGTTTGAGGGAAAAGTAG |
| TRV2-R | CAAAAGACTTACCGATCAATC |
| 18s-F | CCAGGTCCAGACATAGTAAG |
| 18s-R | GTACAAAGGGCAGGGACGTA |
| qDcaCHS-F | ACGCTTTGTATCAGGCGGAC |
| qDcaCHS-R | AGCGCTTTCTGATCATCGATTT |
| qDcaCHI-F | TGCCATTAACGGGTAAACAGTAC |
| qDcaCHI-R | GAATGAGAGAAGACGATGGAAGTG |
| qDcaF3H-F | GTGGGGGATTTTTCAGGTAGTG |
| qDcaF3H-R | CTTCCCTTCCTTCATCGCATAC |
| qDcaDFR-F | CCAGTAGTGGTGACTGGAGCC |
| qDcaDFR-R | AGGCTTCACTTTCTTGAGATTTGTT |
| qDcaANS-F | TACTGCAAGAGTAGAGATCCTCGC |
| qDcaANS-R | GATGTTGAGGGCGTCTACGATAT |
| qDcaWRKY2-F | ATGGCTGGGATGGATGACG |
| qDcaWRKY2-R | GGCTCTGATAGAGAACCATCGTTT |
